# Augmented reality for minimally invasive spinal surgery

**DOI:** 10.3389/fsurg.2022.1086988

**Published:** 2023-01-27

**Authors:** Fedan Avrumova, Darren R. Lebl

**Affiliations:** Department of Spine Surgery, Hospital for Special Surgery, New York, NY, United States

**Keywords:** AR, MISS, fusion, pedicle screw, robotic-assisted navigation

## Abstract

**Background:**

Augmented reality (AR) is an emerging technology that can overlay computer graphics onto the real world and enhance visual feedback from information systems. Within the past several decades, innovations related to AR have been integrated into our daily lives; however, its application in medicine, specifically in minimally invasive spine surgery (MISS), may be most important to understand. AR navigation provides auditory and haptic feedback, which can further enhance surgeons’ capabilities and improve safety.

**Purpose:**

The purpose of this article is to address previous and current applications of AR, AR in MISS, limitations of today's technology, and future areas of innovation.

**Methods:**

A literature review related to applications of AR technology in previous and current generations was conducted.

**Results:**

AR systems have been implemented for treatments related to spinal surgeries in recent years, and AR may be an alternative to current approaches such as traditional navigation, robotically assisted navigation, fluoroscopic guidance, and free hand. As AR is capable of projecting patient anatomy directly on the surgical field, it can eliminate concern for surgeon attention shift from the surgical field to navigated remote screens, line-of-sight interruption, and cumulative radiation exposure as the demand for MISS increases.

**Conclusion:**

AR is a novel technology that can improve spinal surgery, and limitations will likely have a great impact on future technology.

## Introduction

**Figure 1:**
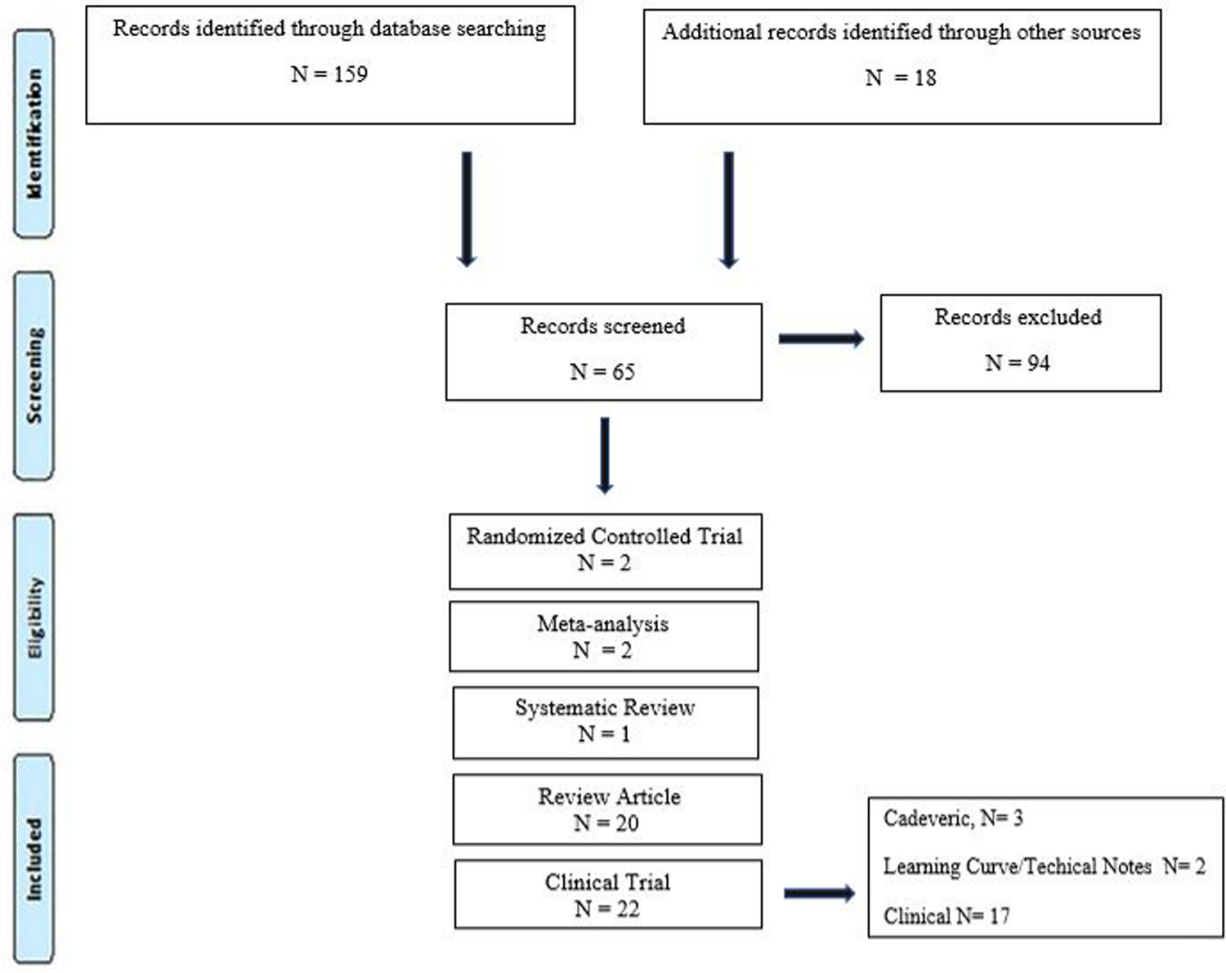
PRISMA flow diagram for the systematic review detailing the database search.

Augmented reality (AR) is an emerging technology that can overlay computer graphics onto the real world and enhance visual feedback from information systems (1). Based on advancements in optics, sensing, and computer systems, AR allows researchers to expand its applications (1). Modern-day AR systems have been integrated into our daily lives, including but not limited to social media, video games, retail, television broadcasting, wearable accessories, education, and in medicine, specifically in minimally invasive spine surgery (MISS) (Figure 1).

Over the past several decades, AR has grown to be an area of interest across many surgical fields, especially with its role in spinal surgery. Recently, AR systems have been implemented in treatment of degenerative cervical, thoracic, and lumbar spine diseases (2, 3). Studies have described AR as an alternative to current approaches such as traditional navigation, robotically assisted navigation (RAN), fluoroscopic guidance, and free hand, as it is capable of projecting patient anatomy directly onto the surgical field (2, 4–10). Therefore, it eliminates surgeon attention shift from the patient to the monitor for guidance (11, 12), line-of-sight interruption on live computer navigation, which may result in the loss of live navigation (13), and cumulative exposure to ionizing radiation as patients’ demand for MISS continues to grow (14).

Although AR is a novel technology that may distinguish itself from other state-of-the-art navigation systems, it is still in its nascency, and several limitations are important to recognize, such as mechanical and visual discomfort (15) and delays in the surgical learning curve, as it may be dependent on a generation of surgeons who grew up playing video games (16–18). Lastly, this is still a new field in research, and while pedicle screw insertion can be guided with AR, there has still yet to be an established system for pedicle screw accuracy (19).

The purpose of this article is to address previous and current applications of AR, AR navigation in MISS, limitations of today's technology, and future areas of innovation.

## Methods

A systematic review was conducted using the PRISMA (Preferred Reporting Items for Systematic Reviews and Meta-Analyses) guidelines.

### Search strategy and data inclusion

Scientific evidence published from May 1997 to August 2022 in PubMed, Medline, and Google Scholar scientific databases was recorded. Keywords *augmented reality*, *robotics-assisted surgery*, *navigation*, *heads-up display*, *minimally invasive surgery*, *spine surgery*, *pedicle screw*, and *accuracy* were used and combined by means of Boolean operators AND and OR under English search. Categories were developed to classify studies as either clinical trial (cadaveric, clinical, and learning curve/technical notes), meta-analysis, randomized controlled trials, review articles, systematic review, and additional sources. The criterion for selecting the articles was published or supported by an indexed scientific database (Figure 1).

After performing each search, potentially relevant articles were identified after reading the title and the abstract. The following information was extracted from each included source: authors, year of publication, historical background, supported significant findings in AR and RAN, and possible limitations. At last, the risk of bias and study quality were assessed by both authors (FA and DRL) by eliminating selection bias, detection bias, reporting bias, and other biases. Ethical approval was not applicable for conducting this systematic review and meta-analysis.

## AR: past and current applications

**Figure 2:**
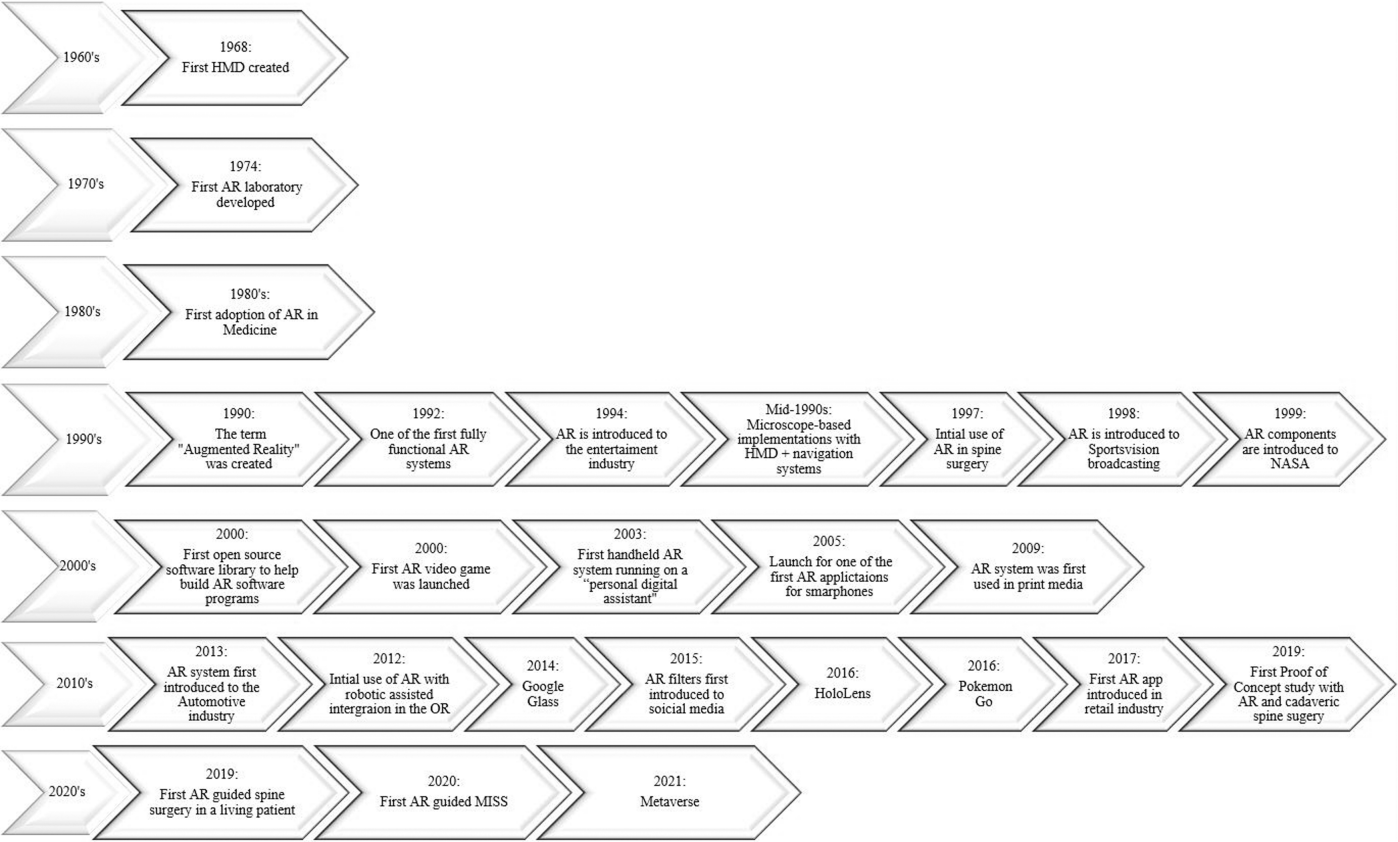
Timeline of AR technology (1960s–present). AR, augmented reality.

AR has become a point of interest in multidisciplinary research fields over the last few decades as it has been used in different applications to enhance visual feedback from information systems (1). Modern-day AR systems have been integrated into our daily lives based on previous systems created decades before (Figure 2). In 1968, the world's first head-mounted display (HMD), known as the “*Sword of Damocles*,” was created by Ivan Sutherland, a Harvard professor and computer engineer (20). The purpose of an HMD was to track the user’s head *via* an ultrasonic position sensor or mechanical linkage and create three-dimensional (3D) lines that appear stationary in the room (21). This allowed users to experience computer-generated graphics that enhanced their sensory perception of the world, which paved the road for AR systems that we currently use today (21). In 1974, a laboratory solely dedicated to AR was created at the University of Connecticut by Myron Kruger, a computer researcher and artist (20, 21). Within the laboratory walls, projections and camera technology were used to emit onscreen silhouettes surrounding users for an interactive experience (20, 21).

### AR in flight

Throughout the 1970s and 1980s, Myron Krueger, Dan Sandin, Scott Fisher, and others experimented with many concepts of mixing human interaction with computer-generated overlays on video for interactive art experiences (22). In 1990, the term “*Augmented Reality*” was coined by Thomas Caudell and David Mizell, Boeing researchers (20, 22). Their technology assisted airplane factory workers as AR managed to display wire bundle assembly schematics in a see-through HMD (22). Around the same time, AR was implemented into different fields of independent research, which led to the creation of one the first fully functioning AR systems known as the “*Virtual Fixture*” by Louis Rosenburg, a researcher in the U.S. Air Force Armstrong's Research Lab (20). This system permitted military personnel to virtually control and guide machinery to perform tasks like training their U.S. Air Force pilots on safer flying practices. In 1999, the first hybrid synthetic vision system was created by NASA for their X-38 spacecraft. This form of AR technology displayed map data on the pilot's screen, which aided with better navigation during flights (20).

### AR released to the public

Components of AR were then later introduced to the public, particularly in entertainment, television, games, social media, and wearable devices. AR made its first debut in the entertainment industry in 1994 by writer and producer Julie Martin. Martin brought AR to her theater production titled “*Dancing in Cyberspace*,” which featured acrobats dancing alongside projected virtual objects on the physical stage (20). In 1998, AR was introduced to Sportsvision broadcasts to draw the First and Ten Yard line in an NFL game (20, 21). By the dawn of the new century, the first open-source software library was created to help build an AR software program known as “*ARTool Kit*,” and the first AR game *AR Quake* was launched (20, 23, 24). The player users wore an HMD and backpack containing a computer and gyroscopes to be able to walk around in the real world and play *Quake* against virtual monsters (24).

By 2003, the first handheld AR system running autonomously on a “personal digital assistant” was created and became the precursor for today's smartphones (22). In 2005, one of the first face-to-face collaborative AR applications developed for mobile phones was created, known as “*AR tennis*,” by Nokia (23, 25). By 2016, Niantic and Nintendo launched *Pokémon Go*, which became a popular location-based AR game (20). This put AR on the map for the general masses leading to the development of similar games (20). Not only has AR been implemented into technology but it also has managed to have a grip on print media. In 2009, Esquire Magazine used AR for the first time; when readers scanned the cover, the AR-equipped magazine featured a celebrity speaking to readers (20).

By 2013, AR was introduced to the automotive industry as Volkswagen introduced the Mobile Augmented Reality Technical Assistance application (20). This was a groundbreaking adaption of AR because this system gave technicians step-by-step repair instructions within the service manual and would be applied to many different industries to align and streamline processes (20). That following year, Google released *Google Glass* to the public, a pair of AR glasses that users could wear for an immersive experience, where users wore the AR technology and communicated with the Internet *via* natural language processing commands and could access applications like Google Maps, Google+, Gmail, and more (20). Two years later, Microsoft created its own version of wearable AR technology known as *HoloLens*, which is more advanced than *Google Glass* as the headset runs on Windows 10 and is essentially a wearable computer. It also allows users to scan their surroundings and create AR experiences (20). Later on, a newer iteration known as the *HoloLens 2* headset was created to target business and medicine (20).

Social media and retail industry started to later apply AR software to their products targeting everyday consumers. In 2015, Snapchat introduced its “*Lenses*” feature by overlaying various filters onto the camera's field of view to alter the perception of the user—Instagram and Facebook followed suit in 2017, applying similar software (20, 26, 27). In the same year, AR was introduced into the retail industry by IKEA, when it launched its AR app called *IKEA Place*, ultimately allowing customers to virtually preview their home decor options before actually making a purchase (20). By 2021, Meta, otherwise known as Facebook, released the first hyper-real alternative online virtual world that incorporates AR, virtual reality, and 3D holographic avatars, video, and other means of communication known as the “*Metaverse*” (28).

### Applications of AR in surgery

One of the first AR adopters in medicine implemented AR in cranial neurosurgeries in the 1980s (15). Attempts to merge image injection systems in operating microscopes led to microscope-based implementations with integrated HMD and navigation systems in the mid-1990s (29). In 1997, Peuchot et al. first described a system known as “Vertebral Vision with Virtual Reality,” which allowed for fluoroscopy-generated 3D transparent visions of the vertebra to be superimposed onto the operative field (30, 31). From there, many generations were developed throughout the years as AR was able to blend intraoperative imaging or models with the surgical scene (31). At the time, this was an innovative approach as it aided in watching vertebral displacements occur without the distraction of referring to a monitor and had the potential to lower exposure to ionizing radiation (31). Studies such as Theocharopoulos et al. reported that levels of ionizing radiation in intraoperative fluoroscopy in spinal surgery are considerably higher than those in other subspecialties, and AR systems report a significantly lower dosage of radiation (31, 32).

In 2012, Leven et al. and later Schneider et al. proposed “flashlight” visualization to overlay the intraoperative ultrasound image onto a 3D representation of the imaging plane in the stereo view of the console, which then led to a drop-in tool for registering transrectal ultrasound images with laparoscopic video (21). Throughout the years, as AR technology was made available to the public and with the release of *HoloLens* in 2016, a newer iteration known as the *HoloLens 2* headset was created where surgeons can implement this technology in the operation field (20, 33). This headset fits over the surgeon's head and displays transparent images that hover in the surgeon's field of vision. The application aligned images of the patient's anatomy with the real-life view. The surgeon then can walk around the patient, viewing three-dimensional holographic images of internal structures from different vantage points (33). Surgeons may also use voice commands or hand gestures to enlarge images or move information around. Even the patient's vital signs can be projected onto the field of vision (33).

**Figure 3:**
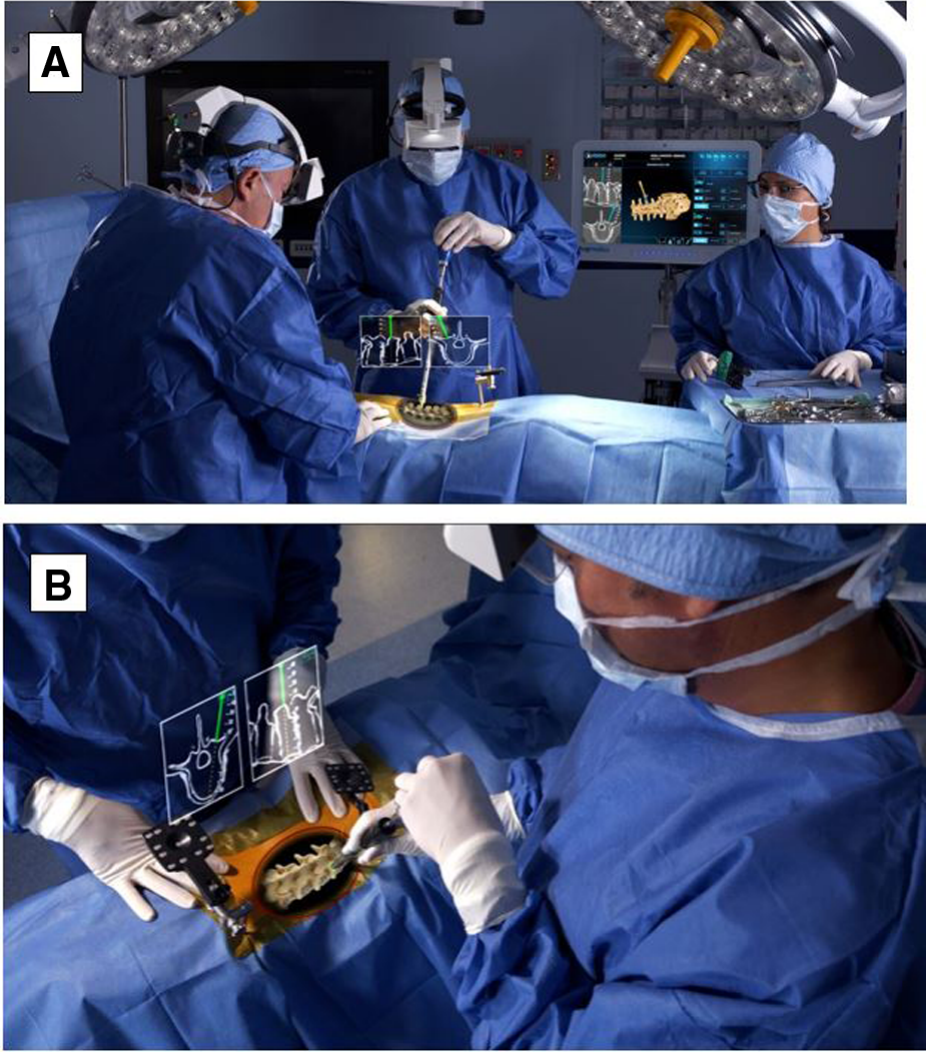
(**A**) HMD projects a 3D visualization of the surgeon's retina. (**B**) Surgeon sees a 3D image of the patient's spine with two-dimensional CT images of the instruments’ path and trajectory while looking directly at the surgical field. HMD. head-mounted display; 3D, three-dimensional; CT, computed tomography. Courtesy of Augmedics.

By 2020, the first AR-guided spine surgery in a living person was performed with the Xvision system by Augmedics at John Hopkins University by Dr. Timothy Witham (34, 35). The first procedure was performed on June 8, 2020, where six screws were used during a spinal fusion surgery to fuse three vertebrae to relieve the patient from chronic back pain (34). The second surgery was performed on June 10, 2020, where surgeons removed a cancerous tumor from the spine of a patient (34). However, the first proof-of-concept study with AR-assisted pedicle screw insertion and cadavers was published in 2019 by Dr. Frank Phillips at Rush University. Within that following year, Phillips performed the first AR-guided MISS by implementing the same Augmedics system (19, 36). Phillips was able to perform a lumbar fusion with spinal implants on a patient with spinal instability (36). During the MISS procedure, the headset projected a 3D visualization of the navigation data onto the surgeon's retina. This allows the surgeon to see a 3D image of the patient's spine with the skin intact and two-dimensional (2D) computed tomography (CT) images of the instruments’ path and trajectory while looking directly at the surgical field (36) (Figure 3).

**Figure 4:**
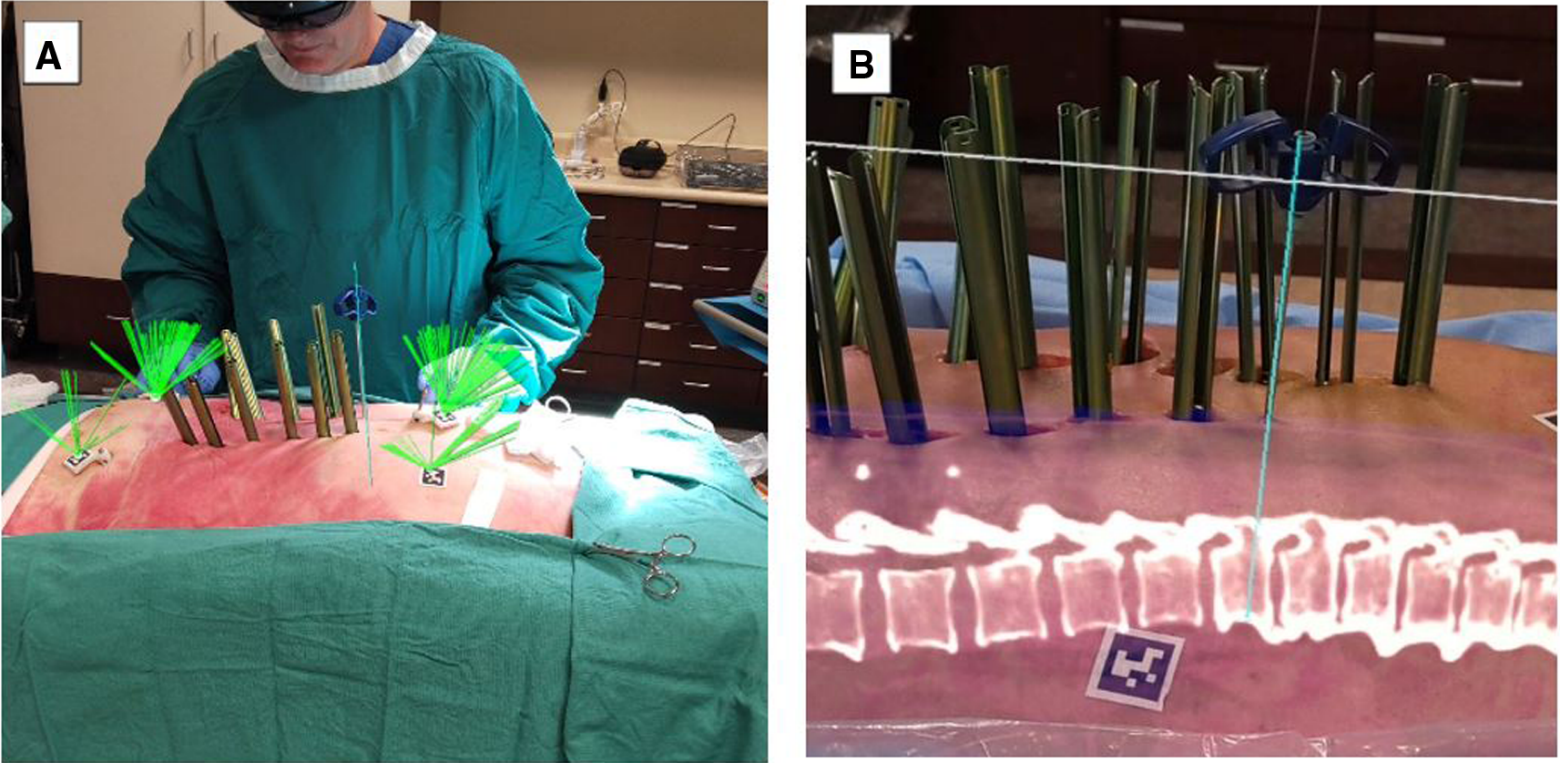
MISS navigation procedure utilizing VisAR technology. (**A**). The green ray (usually not seen) continuously monitors the centrum of each AprilTag for ongoing adjustment of the registration, if required. AprilTags are adhered to the skin and also placed on platforms stabilized by bone pins. The Jamshidi needle is aligned with the virtual needle/pathway and has been inserted percutaneously. (**B**). Lateral view of a MISS procedure in progress under VisAR navigation. Note the Jamshidi needle that has successfully penetrated the underlying pedicle. The center of the needle has been extracted, and a K-wire has been inserted for guidance of a cannulated screw. An optical fiducial (AprilTag) appears below the vertebrae. MISS, minimally invasive spine surgery. Courtesy of VisAR.

Currently, wearable devices, such as HMD, have been commonly used to display AR views for ease and speed and to accomplish feasibility, accuracy, and safety in workflows from open cases to MISS (14, 19). Current platforms include key features such as reducing attention shift from the surgical field to monitors, line-of-sight interruption, and cumulative exposure to ionizing radiation. Newer generations of AR systems, such as Augmedics and VisAR, have the capability to overlay virtual bony structures and preplanned screw trajectories on patients in the OR, enabling real-time feedback of all instruments in space in relation to anatomical structures (36) (Figures 3, 4). This removes the need for surgeons to avert their eyes to a screen and the traditional utilization of markers and tracking cameras, as AR systems are designed to align the hands and eyes of the surgeon (37, 38). Thus, real-time 3D capabilities allow the surgeon to “augment” the quantity of information that can be inferred by the sole surgeon's eyes (3, 39) (Figures 3, 4).

### Pedicle screw accuracy in current AR platforms

Since accomplishing the first AR-guided surgeries in MISS surgeries, current platforms are assessing pedicle screw accuracy in MISS procedures. Studies have reported that Xvision by Augmedics has pedicle screw accuracies greater than at least 97.7% (40). Felix et al. compared pedicle screw accuracies between open and MISS procedures as both were guided by the AR system, VisAR (14). A total of 124 pedicle screws were inserted with VisAR navigation with 96% accuracy (Gertzbein–Robbins grades A and B), reporting that AR is an emerging technology can be highly accurate for both surgeries (14).

## Discussion

### The additive value of AR in RAN

With the emergence of AR and its multidisciplinary applications, AR is reported to assist RAN in more complex surgeries (41). Miller et al. reported prior AR work done on the DaVinci robot to show a 3D model of the patient's prostate superimposed on the intraoperative view (41, 42). Forte et al. explored alternative uses and interaction methods of AR and RAN and presented a robot-independent hardware and software system that provides four intuitive AR functions through computer graphics and vision (41). These functions can bring additional visual information into the surgeon's view, and the other functions leverage computer vision to provide more sophisticated computational capabilities (41). This relies only on vision rather than robotics to provide precise visual alignment between AR markers and the instrument and to avoid lengthy calibration procedures that are challenging for nontechnical personnel to perform (41, 43).

Although AR may bring an additive value to RAN, established RAN workflow for pedicle screw instrumentation may be subject to concern. Over the past decade, RAN has been implemented in surgeries as a practical tool to advance the field of MISS (44, 45). The first robotic-assisted system for adult spine surgery received U.S. Food and Drug Administration (FDA) clearance in 2004 (37). Robotic systems with integrated surgical navigation have the potential for improved accuracy, shorter time-per-screw placement, less fluoroscopy/radiation time, and shorter hospital stay than freehand (FH) techniques (46). Since then, newer robotic devices have been developed and cleared by the FDA for use in spine surgery (28). The RAN workflow for pedicle screw instrumentation can be simplified into three steps: preoperative planning, intraoperative registration, and robot-guided screw placement (47).

**Figure 5:**
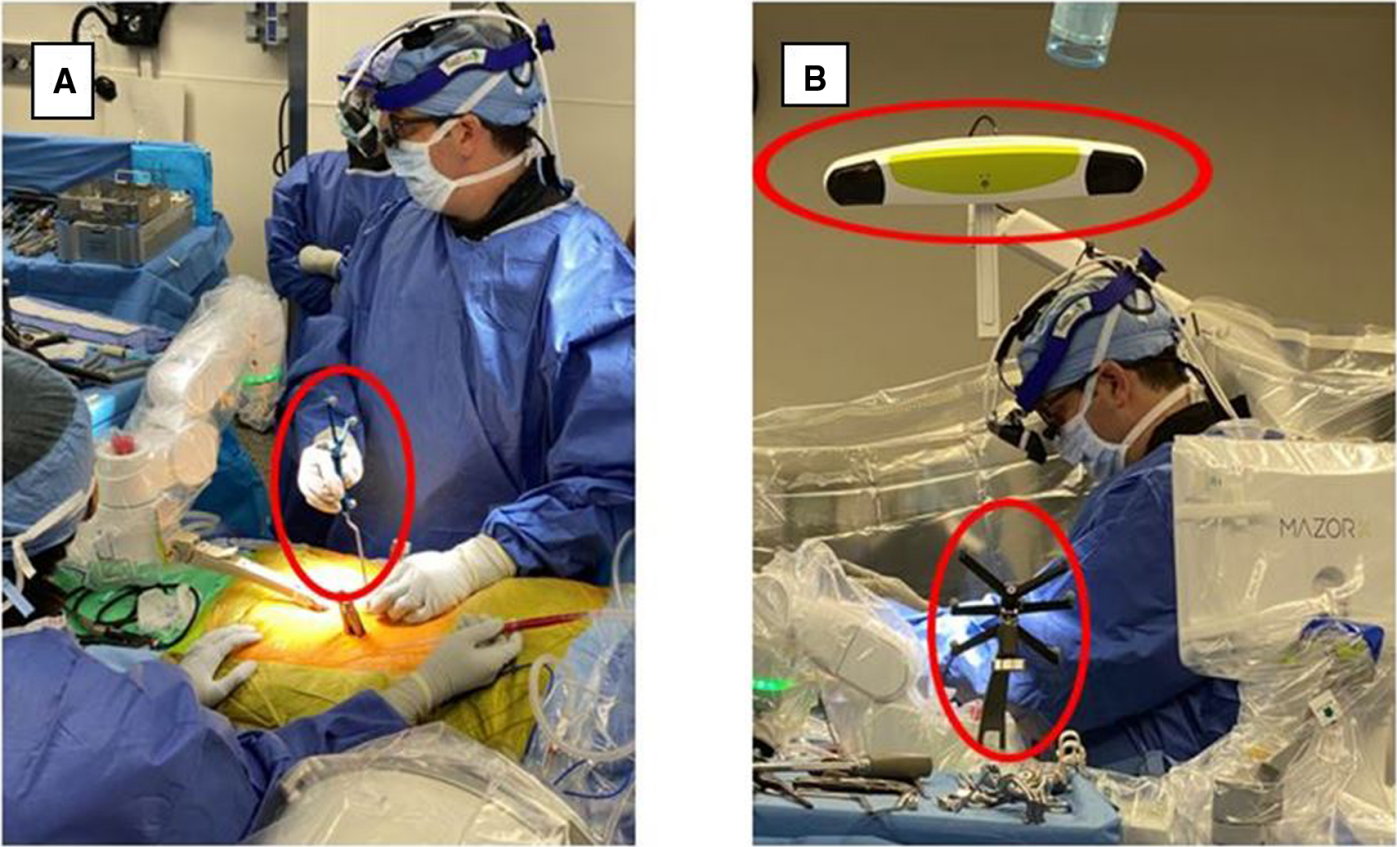
Ran registration process, all required surgical instrumentation was registered and verified *via* 3Define cameras. RAN, robotically assisted navigation.

**Figure 6:**
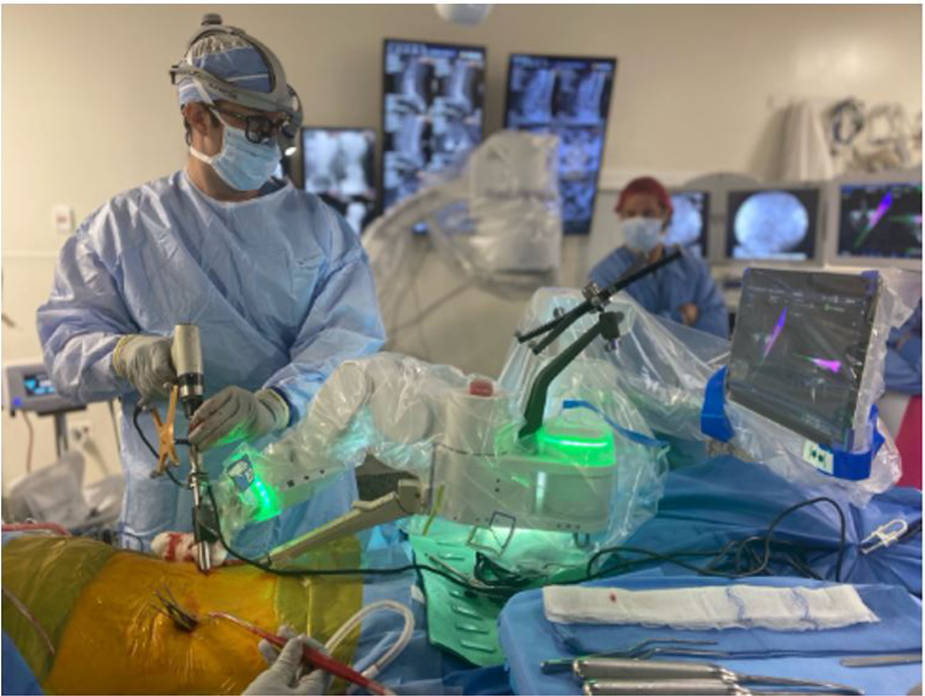
RAN surgeries incorporating registered navigated instrumentation, drill guides, and surgical monitors. Drill guide during surgical procedure combined with real-time visual feedback from the surgical navigation monitors. The navigation monitor shows real-time visual feedback based on the positioning of the navigated instrument. RAN, robotically assisted navigation.

First, preoperative CT imaging is loaded onto the robotic planning software (48). Preoperative planning for screw insertion is carried out on robotic software, including the determination of the screw entry point, the size of screws, and the trajectories planned in axial and sagittal views preoperatively. Next, intraoperative registration is performed as a robotic mount is attached to the reference posterior superior iliac spine (PSIS) or spinous process reference clamp and ends with confirmation of fluoroscopic imaging, which is colocalized with the software planning template. During this step, all required surgical instrumentation can be registered and verified *via* 3Define cameras (Figure 5). At last, robotic-guided screw placements are initiated when a robotic arm is positioned over a single planned pedicle and end when the robotic arm is retracted following screw insertion (48) (Figure 6).

However, concerns related to RAN include surgeon attention shift from the surgical field to navigated remote screens, line-of-sight interruption, and cumulative radiation exposure as the demand for MISS increases.

### Surgeon attention shift from the surgical field to navigated remote screens

Conventional navigated methods include a shift in surgeons’ attention from the surgical field to a navigated remote screen. Similar to manual navigated systems, RAN requires the surgeon to observe the navigated screw insertion trajectory on a remote screen, making RAN just as susceptible to similar attention shift errors (19). Molina et al. reported issues that arose with attention shift, including preoperative errors caused by errors in preoperative planning, soft tissue pressure on instrumentation, a shift in the entry point and instrument positioning, and morphology of the starting point causing skive (19, 48).

Attention shifts have been shown to negatively impact both cognitive and motor tasks and add time to performing the task (11, 12). Goodell et al. evaluated laparoscopic surgical simulation tasks designed to replicate the levels of cognitive and motor demands in surgical procedures and found a 30%–40% increase in task completion time in the distracted vs. undistracted condition (49). In addition to that, Léger et al. reported the number of attention shifts needed to perform a simple surgical planning task using both AR and conventional navigation and found that AR systems (mobile and desktop) were statistically different from the conventional navigation systems but were not statistically different from one another (11). The errors associated with attention shift can be removed by directly projecting the navigation guidance onto the surgical field, allowing surgeons to keep their attention on the surgical field (19).

Furthermore, Léger et al. described that when AR is utilized for procedures, the attention of the subject remains almost the whole time (90%–95%) on the guidance images; however, other guidance systems split the attention almost 50/50 between the patient and the monitor (11). Additionally, the ratio of time based on looking at the screen to total time taken may give an estimate of the user's confidence in what one is doing; therefore, the higher ratios obtained for AR systems may indicate that AR gives users more confidence that they are correct with respect to the data presented (11).

### Line-of-sight interruption

Aside from attention shift, another common limitation of RNA guidance is line-of-sight interruption (19). During procedures, live computer navigation is interrupted by an obstacle that blocks the visualization of tracking markers by a remote tracking camera, resulting in the loss of live navigation until the obstruction is resolved (19). This is a common limitation as it may increase the operative time and decrease the accuracy (14). To combat such barriers, newer AR systems have developed an adjustable headset, a built-in tracking system, and an integrated headlight that projects AR onto a small optical display or directly onto the surgeon's retina. This form of AR is known as AR-HMD (Figures 3A, 4A) (14).

Patient anatomy is obtained by automated segmentation of the intraoperative cone beam CT scan. The surgeon is also able to see 2D sagittal and axial projections within the headset (Figures 3B, 4B). The headset projects holograms directly to the surgeon's retina, allowing for 3D superimposition of the anatomy over the real spine, and clinical accuracy is then measured shortly after (19). Studies such as Molina et al. reported the first cadaveric experience employing an AR-HMD that provides the ability to insert 120 pedicle screws and found overall insertion accuracies of 96.7% and 94.6% using the Heary–Gertzbein and Gertzbein–Robbins grading scales, respectively (19). Similarly, Liu et al. published a study with 205 consecutive pedicle screw placements recorded in 28 patients (49). Screw placement accuracy was graded only with the Gertzbein–Robbins scale and reported a 98.0% accuracy, in line with the reported accuracy of navigation (49, 50). Although AR navigation may present high accuracy based on nonstandardized grading scales, the initial use of the system may result in sensory overload, and a learning curve is to be expected (14). Nonetheless, AR-HMD maintains a major advantage of minimizing line-of-sight interruption, as it can be used for both open surgery and MISS (14, 35, 51).

### Cumulative radiation exposure as the demand for MISS increases

Radiation exposure for spine surgeons, OR staff, and patients has been a concern for many years (14). According to Bratschitsch et al., there has been a more than 600% increase in the use of radiation for diagnostic procedures in the United States since the 1980s (52). A 2014 report published an increased risk of cancer by up to 13% among members of the Scoliosis Research Society, advising robust safety measures for staff and spine surgeons (53).

Over the years, intraoperative imaging and surgical approaches have evolved with spine surgery. This generally guided surgeons to less invasive approaches, such as MISS techniques. Stanford Medicine reported that MISS techniques and RAN integration have shown shorter operating times and reduced pain and discomfort in patients (54). Furthermore, a meta-analysis comparing percutaneous and open pedicle screw placement for thoracic and lumbar spine fractures suggested that MISS is a superior treatment approach for pedicle screw placement (55). However, this technique calls for more imaging guidance and ultimately increases radiation exposure because fluoroscopy remains necessary to confirm vertebral levels, check spinal alignment, and guide implant placement (56, 57).

Technologies to reduce intraoperative radiation have real potential to impact long-term risks. As wearable and independent of navigation monitors, AR guidance can avoid attention shift, decrease OR clutter, and does not use or require ionizing radiation for surgical guidance. Studies such as Felix et al. have documented recent advancements in AR as a “paradigm shift” in its application in a variety of surgical fields, including orthopedics, neurosurgery, and spine surgery (14).

## Technical pearls associated with AR

Although AR is a novel technology that may distinguish itself from other state-of-the-art navigation systems, it is still in its nascency and several technical limitations are important to recognize. First, mechanical and visual discomfort may arise from AR devices such as AR headwear (AR-HMD) (15). Furthermore, visual discomfort, visual obstruction of anatomy by holographic images, and the need for intraoperative, rather than preoperative, CT scans for registration limit its applications (15). The surgeons’ initial experience with an AR system may be disorienting mostly due to factors such as mixing real visual input with holographic data projected onto the surgeons’ retina, resulting in sensory overload, and a learning curve is to be expected (50).

Delays in the surgical learning curve associated with AR may be attributed to a surgeon's ability to adapt and may depend on a generation of surgeons who grew up playing video games (16–18). Spine surgery learning curves can replicate in-line maneuvers while placing instrumentation, as well as adopting and developing proper technique while using image-guided technology. Rosser et al. described a correlation between faster completion and reduced errors in laparoscopic surgeries when the surgeons' background consists of more than 3 h per week of video game play (16, 58).

Lastly, there is a limited amount of literature in regard to AR assistance with workflow related to pedicle screw placement and no universally accepted standard method to grade screw accuracy and/or safety (14). Common systems such as Gertzbein and Robbins classification are typically utilized; however, they only measure medial, superior, and inferior cortical pedicle breaches and partial lateral breaches (such as in the in/out technique) (14). Heary et al. recognized thoracic pedicle screw accuracy grading without considering the direction of the breach being inadequate, specifically in the case of deliberate thoracic pedicle screw in–out–in trajectories (59). Thus, further studies need to be reported to find a standardized approach as AR technology is developing rapidly.

## Future perspectives

With current advancements in the last several years, the use of AR in spinal surgery promises an exciting future. Future updates/versions of AR technology and wearable devices may improve the ability to manipulate display and radiographic scans, enhance surgeon's alertness when approaching critical structures *via* hepatic feedback, and reduce intraoperative complications (15). In addition to that, it has the potential to serve as a critical tool for preoperative planning and an educational tool for future medical students, residents, and fellows.

Furthermore, several RAN and AR systems for spine surgery have been released and tested in numerous clinical studies (42). Although RAN does have many advantages, such as lower risk of neurovascular damage, reoperation rates, postoperative infections, time for ambulation, and length of stay, and is typically studied for its pedicle screw accuracy rates compared to FH, many limitations are of concern (42). Currently, there are several limitations, such as complications with hardware or software failure, a demanding learning curve, no uniform consensus regarding operative time, cannula misplacements, and skiving off the drilling tip onto the pedicle surface due to the morphology of the starting point (48). Therefore, newer technologies such as AR systems have been reported to be more competitive in terms of an easier approach, setup, and real-time patient positioning monitoring for correction of surgical plans (42), as well as having the ability to address concerns associated with RAN as previously mentioned.

The role of AR in spine surgery is a rapidly evolving field where new technology and surgical techniques can help maximize surgical efficiency, precision, and accuracy. With collaborations from clinicians, engineers, and video game designers, this technology can profoundly improve components of MISS. Thus, it is conceivable that within the next few years surgeons will be wearing AR glasses during patient consultation, rehabilitation, training, and surgery. Such advancements, among others, will continue to drive the value of AR 3D navigation in MISS.
